# The Ticking Clock: Differential Time‐Dependent Deterioration Between Washed and Thawed Sperm

**DOI:** 10.1111/andr.70150

**Published:** 2025-11-24

**Authors:** Adiel Kahana, Emily Hamilton, Noga Fuchs Weizman, Sandra E. Kleiman, Shlomit Shabat, Foad Azem, Shimi Barda

**Affiliations:** ^1^ The Institute for the Study of Fertility and Racine IVF Unit Lis Maternity Hospital Tel Aviv Sourasky Medical Center Tel Aviv Israel; ^2^ NY State/American Program Faculty of Medical and Health Sciences Tel Aviv University Tel Aviv Israel; ^3^ Faculty of Medical & Health Sciences Tel Aviv University Tel Aviv Israel; ^4^ Israel Academic College Ramat Gan Israel

**Keywords:** DNA fragmentation, intrauterine insemination, sperm cryopreservation, sperm washing, time‐dependent deterioration

## Abstract

**Background:**

Cryopreservation is widely used in assisted reproductive technologies. While fresh sperm undergoes gradual time‐dependent deterioration, it remains unclear whether thawed sperm exhibits a more accelerated decline.

**Objectives:**

To directly compare the rate of deterioration in sperm motility, vitality, and DNA fragmentation between fresh washed and thawed sperm samples over time.

**Materials and Methods:**

This prospective study included semen samples from 50 males. Samples were split into two groups: Washed (freshly washed sperm) and Thawed (washed, cryopreserved for at least 2 weeks, and thawed). Sperm parameters, including motility, vitality, and DNA fragmentation index (DFI), were assessed immediately after processing (Time 1) and again after 75 min incubation at room temperature (Time 2). Additionally, control experiments tested whether cryoprotectant exposure alone could account for deterioration by comparing washed samples incubated with washing versus freezing medium (10 patient samples), and by assessing post‐thaw washing (10 donor samples).

**Results:**

Total sperm motility declined significantly more in thawed samples (29 ± 16%) compared to fresh washed samples (17 ± 9%, *p* < 0.0001). Vitality similarly deteriorated more in thawed samples (21 ± 14%) versus fresh washed samples (7 ± 5%, *p* < 0.0001). DNA fragmentation increased significantly only in thawed samples (*p* = 0.0331), reaching clinically critical levels (mean DFI 34 ± 13% at Time 2), compared to fresh samples which remained within normal range (12 ± 5%). Motility grade transitions differed markedly, with thawed samples showing direct transitions from Grade A motility to immotility, unlike fresh washed samples, which transitioned gradually from Grade A to Grade B. In additional control experiments, cryoprotectant exposure alone did not induce deterioration, and post‐thaw washing did not improve metrics.

**Discussion:**

Thawed sperm exhibited accelerated deterioration across all measured parameters, highlighting cumulative stress from cryopreservation. The rapid decline underscores the need to minimize the interval between thawing and insemination.

**Conclusion:**

Thawed spermatozoa demonstrate significantly greater susceptibility to time‐dependent deterioration compared to fresh washed samples, advocating for immediate use post‐thaw to optimize reproductive outcomes.

## 1 Introduction

The cryopreservation of human spermatozoa has been a fundamental component of assisted reproductive technologies since its introduction in the 1960s [1]. This technique has evolved significantly over the decades, with major advancements in cryoprotectants, freezing protocols, and thawing procedures [2, 3]. Cryopreserved sperm is widely used in assisted reproductive technologies, including intrauterine insemination (IUI) and in vitro fertilization (IVF), serving diverse patient populations [4]. In addition, sperm cryopreservation plays a crucial role in fertility preservation for patients undergoing potentially sterilizing medical treatments, such as chemotherapy, radiotherapy, or surgery [5]. Thus, maintaining sperm quality throughout the cryopreservation process is of paramount importance.

Time‐dependent degradation is a well‐documented phenomenon in fresh sperm samples. Previous studies have reported a 5%–10% decline per hour in sperm motility post‐ejaculation [6]. This deterioration has been attributed to multiple factors, including oxidative damage from reactive oxygen species (ROS), bacterial contamination, and energy depletion due to ongoing metabolic activity [7].

While the temporal degradation of fresh sperm has been extensively studied, limited data exist on how thawed sperm behaves over time. The freezing‐thawing process is known to induce multiple changes in sperm quality [5, 8]. Specifically, it has been shown to cause membrane damage, leading to reduced motility and vitality [9], as well as increased DNA fragmentation and apoptosis in sperm cells [10, 11, 12, 13]. The underlying mechanisms of cryodamage include cold shock, osmotic stress, and oxidative stress [14].

A recent study [15] investigated the impact of incubation time and repeated freeze‐thaw cycles on thawed sperm quality. The study reported a significant decline in sperm parameters over time post‐thawing, with a more pronounced decrease at 180 min compared to 90 min. This finding is clinically relevant, particularly in IUI procedures conducted off‐site, where sperm samples are prepared in the laboratory, but insemination occurs only after the patient arrives at a remote clinic, often resulting in considerable delays.

However, while this study demonstrated time‐dependent deterioration in thawed sperm, it primarily focused on the effects of repeated freeze‐thaw cycles rather than a direct comparison between washed and thawed sperm over time. Thus, the isolated effect of time on thawed sperm, independent of refreezing, remains poorly understood.

Given the widespread clinical reliance on thawed sperm in procedures such as IUI and IVF, understanding whether thawed spermatozoa undergo accelerated deterioration compared to fresh samples during incubation is critical. This knowledge will inform laboratory protocols, optimize timing of insemination, and potentially improve reproductive outcomes. Thus, the current study aims to directly compare the rate of deterioration of thawed versus fresh washed sperm samples over time, providing valuable insights into the optimal handling of cryopreserved sperm.

## 2 Methods

### 2.1 Specimens

This prospective study was conducted at the Male Fertility and Sperm Bank Unit between November 2020 and August 2025. Specimens were collected from male participants who visited the clinic during this period. Participants were enrolled consecutively as they presented to the clinic for routine semen analysis. All participants provided written informed consent prior to enrollment. Men with a history of chemotherapy or those providing samples less than 1 mL in volume were excluded from the study. In addition, for the control experiments, cryopreserved donor samples from the sperm bank were utilized following the same inclusion and exclusion criteria. The study protocol received approval from the Institutional Review Board (approval number: 0145‐18).

### 2.2 Study Design

Following collection, semen samples were allowed to liquefy for 30 min at 37°C before undergoing initial semen analysis. Based on this assessment, a 1 mL aliquot was taken from each sample and divided into two separate test tubes, designated as “Washed Group” and “Thawed Group.”

The Washed Group underwent immediate sperm washing, whereas the Thawed Group underwent sperm washing followed by cryopreservation for at least 2 weeks and subsequent thawing.

Both groups were analyzed at three distinct time points. Baseline analysis (Time 0) was performed immediately following liquefaction, prior to any further processing. Time 1 analysis was conducted immediately after sperm washing (Washed Group) or immediately following thawing (Thawed Group). Finally, Time 2 analysis was performed 75 min after Time 1 to assess potential time‐dependent deterioration.

Semen analysis at all‐time points included sperm motility, vitality, and DNA fragmentation index (DFI). DFI was evaluated in 20 out of 50 samples, selected to represent the overall cohort, given the resource‐intensive nature of the TUNEL assay and the limited availability of specialized equipment.

### 2.3 Power Calculation

To determine the required sample size, an a priori power analysis was conducted using G*Power software [16]. The analysis assumed a statistical power of 0.8, an alpha of 0.05, and a medium effect size (*f* = 0.4), selected based on common practice, as no relevant prior data were available. According to this calculation, a minimum of 50 participants was required to reliably detect the anticipated effects.

### 2.4 Semen Analysis

All semen processing was performed at the Male Fertility and Sperm Bank laboratory, which participates in external quality control programs, including the UK NEQAS External Quality Assessment Schemes for sperm concentration, motility, and morphology.

Semen samples were collected by masturbation into sterile plastic containers after 2–3 days of abstinence. Following liquefaction, fresh semen samples were analyzed according to the World Health Organization laboratory manual for the examination and processing of human semen, sixth edition [17]. Sperm concentration was determined using an Improved Neubauer counting chamber. Sperm motility was assessed under microscopy at 37°C ± 0.5°C by evaluating at least 300 spermatozoa per aliquot, classifying motility using a four‐category scheme: rapid progressive, slow progressive, non‐progressive, and immotile. Motility assessments were performed using a 10 µL drop covered with a 22 × 22 mm coverslip, creating approximately 20 µm depth. Sperm vitality was assessed using eosin‐nigrosin staining at 1000× magnification using oil immersion. Morphology was evaluated by Papanicolaou staining and classified as normal or abnormal according to the strict Kruger criteria [18], also assessed under 1000× magnification using oil immersion. To minimize interobserver variability, all analyses were performed by a single experienced laboratory technician.

#### 2.4.1 Sperm Processing and Cryopreservation

Semen samples were processed according to their designated group. Both the Washed Group and Thawed Group underwent sperm washing using modified Human Tubal Fluid (mHTF) medium (Irvine Scientific, Santa Ana, CA) in a 1:2 ratio, followed by centrifugation at 300 g for 10 min. The washed sperm pellet from the Washed Group was resuspended in 0.3 mL of mHTF and kept at room temperature until analysis.

For the Thawed Group, the washed pellet was resuspended in 0.3 mL of mHTF and carefully diluted by adding an equal volume of TEST‐yolk buffer freezing medium (Irvine Scientific, Santa Ana, CA). The mixture was equilibrated for 20 min at room temperature, then sealed in 0.5 mL straws (Minitube, Tiefenbach, Germany) and cooled in a semi‐programmable freezer (Nicool LM‐10; Air Liquid, Paris, France). The controlled cooling process followed a protocol decreasing from room temperature to −5°C at a rate of 1.7°C/min, then from −5°C to −100°C at a rate of 5°C/min. The straws were then transferred directly to liquid nitrogen (−196°C) for long‐term storage.

Thawing was performed by placing frozen sperm samples on a hotplate at 37°C for 5 min before transferring them to 1.5 mL tubes. The samples were thoroughly mixed to ensure homogeneity and subsequently incubated at room temperature. Sperm parameters were analyzed as previously described.

### 2.5 Assessment of DNA Fragmentation by TUNEL Assay

DNA fragmentation was evaluated using the In Situ Cell Death Detection Kit (Roche, Basel, Switzerland). Semen samples were smeared onto microscope slides and fixed in 4% formaldehyde phosphate‐buffered solution. After TUNEL labeling, nuclei were counterstained with DAPI (Sigma‐Aldrich, St. Louis, MO), and cells were examined using fluorescence microscopy (Olympus Provis AX70, Tokyo, Japan). A total of 300 sperm cells per sample were counted to determine the percentage of TUNEL‐positive cells. Positive and negative controls were included in each assay to ensure experiment quality. Positive control was prepared by incubating samples with DNase I for 10 min before TUNEL labeling, while the negative control was prepared by omitting the enzyme from the TUNEL labeling solution.

### 2.6 Assessment of Cryoprotectant Exposure and Post‐Thaw Washing Effects

To address potential confounding effects of cryoprotectant exposure, two additional control experiments were conducted:

Medium effect analysis (Experiment 1): Fresh semen samples from 10 patients were processed using the standard washing protocol described in Section 2.4.1. Each sample was divided into two equal aliquots: Group A was resuspended in washing medium (mHTF) alone, whereas Group B was resuspended in a 1:1 mixture of washing medium (mHTF) and freezing medium (TEST‐yolk buffer). Both groups were analyzed at Time 1 (immediately after processing) and Time 2 (75 min later) to isolate the effect of cryoprotectant exposure independent of freeze‐thaw damage.

Post‐thaw washing analysis (Experiment 2): Cryopreserved samples from 10 sperm donors were thawed using the thawing protocol described previously and divided into two equal aliquots: Group C left unwashed, and Group D washed immediately post‐thaw to remove the cryoprotectant. Both groups were analyzed at Time 1 (immediately after thawing/washing) and Time 2 (75 min later) to determine whether post‐thaw washing affects time‐dependent deterioration.

### 2.7 Statistical Analysis

Statistical analyses were performed using R software (version 4.4.3) and JMP (version 16). For the primary study, matched‐pairs analysis was conducted using paired *t*‐tests, following verification of variance homogeneity using Levene's test, to compare sperm parameters (total motility, vitality, DNA fragmentation, and motility grades) between two consecutive time points (Wash 1 vs. Wash 2, Thaw 1 vs. Thaw 2). Repeated‐measures analysis employed a linear mixed‐effects model, with time and group type as fixed effects and subject ID as a random effect. Model fit was assessed using the Akaike Information Criterion (AIC), and variance explained was reported as marginal *R* ^2^. For the control experiments, only matched‐pairs analysis with paired *t*‐tests was employed. A significance level of *p* < 0.05 was considered statistically significant. Data are presented as mean ± standard deviation (SD).

## 3 Results

From an initial cohort of 64 male participants enrolled between November 2020 and August 2025, a total of 50 participants were included in the final analysis. Participants were excluded due to a history of chemotherapy treatment (*n* = 5) or insufficient sperm quality or volume below 1 mL (*n* = 9). DFI was measured in 40% of the analyzed samples (*n* = 20). In addition, 20 supplementary samples were analyzed in the control experiments (10 patients for medium effect analysis and 10 donor samples for post‐thaw washing analysis), bringing the total analyzed samples to 70.

### 3.1 Comparison Between Thawed and Fresh Groups

Table 1 presents sperm quality parameters at different time points, including fresh, washed, and thawed samples. Both matched‐pairs analysis and repeated‐measures analysis were used for statistical comparisons, as detailed in Section 2.

**TABLE 1:** Sperm parameters across different time points (Fresh, Washed, and Thawed Groups).

Parameter (Mean ± SD)	Time 0	Wash Time 1	Wash Time 2	Thaw Time 1	Thaw Time 2
Total motility (%)	57 ± 12	61 ± 12	51 ± 12	37 ± 15	27 ± 14
Motility A (%)	33 ± 15	39 ± 15	26 ± 13	21 ± 16	13 ± 13
Motility B (%)	20 ± 8	19 ± 9	19 ± 8	11 ± 7	9 ± 7
Motility C (%)	4 ± 3	3 ± 2	6 ± 2	5 ± 5	5 ± 4
Vitality (%)	81 ± 8	79 ± 6	74 ± 8	59 ± 14	48 ± 16
DFI (%)	7 ± 3	8 ± 4	12 ± 5	23 ± 12	34 ± 13

*Note*: Data are expressed as mean ± standard deviation (SD).

#### 3.1.1 Total Sperm Motility

**FIGURE 1:**
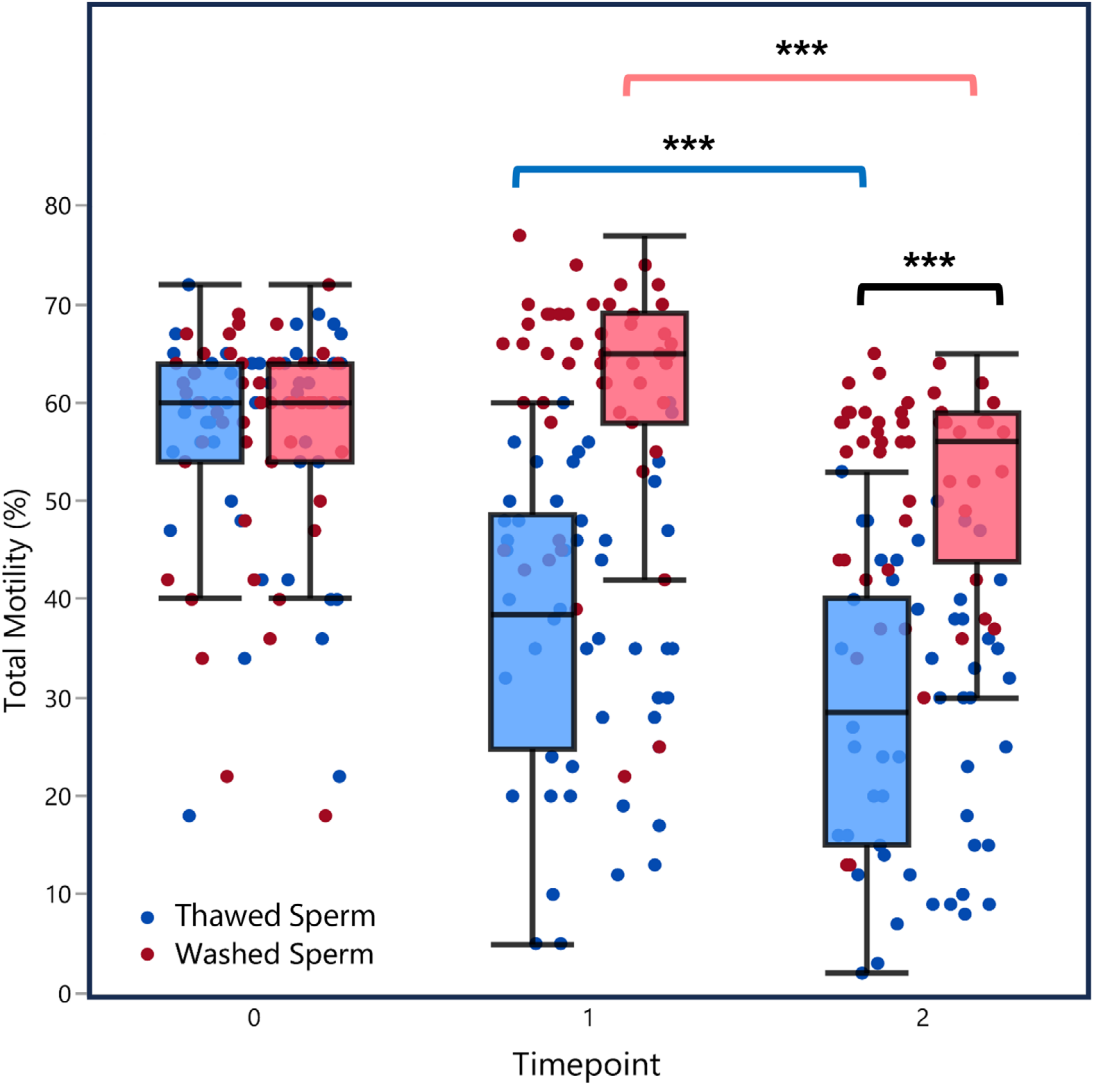
Effect of time on total motility in thawed versus washed sperm samples. Box plots with individual data points (*n* = 50). Blue dots/boxes = thawed sperm, red dots/pink boxes = washed sperm. Time points: 0 = baseline (before processing), 1 = immediately after washing/thawing, 2 = 75 min after washing/thawing. Boxes = interquartile range, line = median, whiskers = non‐outlier range. Statistical comparisons between time points are indicated by brackets with significance levels (^***^ *p* < 0.001).

Matched‐pairs analysis showed a significant deterioration in total sperm motility over time in both Thawed and Washed Groups (*p* < 0.001). The decrease in total motility was significantly more pronounced in thawed samples (29 ± 16%) compared to washed samples (17 ± 9%, *p* < 0.0001). Repeated‐measures analysis further supported this finding, explaining 94.7% of variance due to fixed and random effects (marginal *R* ^2^ = 0.947, *p* < 0.0001). As shown in Figure 1, the rate of motility decline was substantially steeper in thawed samples compared to washed samples.

#### 3.1.2 Sperm Vitality

**FIGURE 2:**
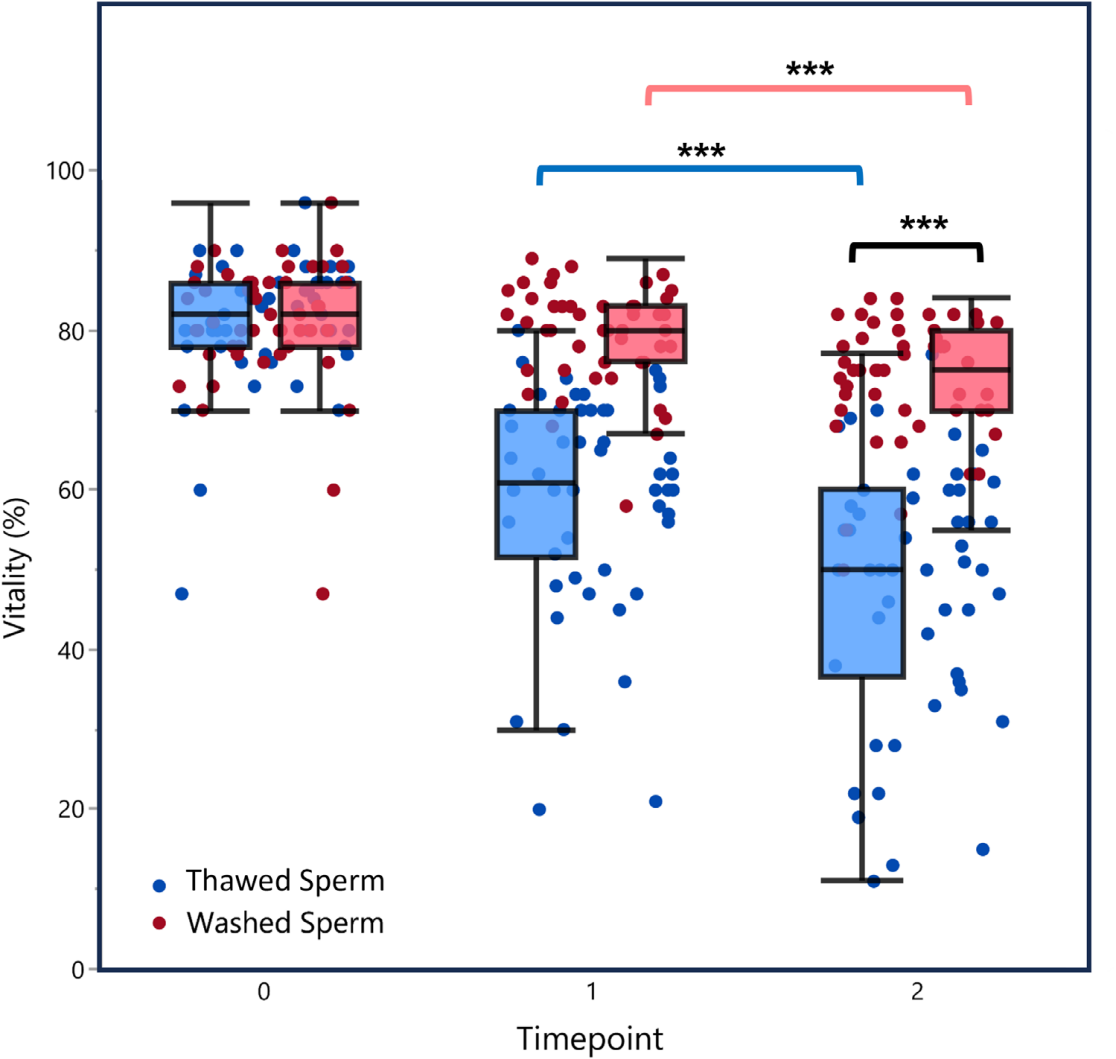
Effect of time on vitality in thawed versus washed sperm samples. Box plots with individual data points (*n* = 50). Blue dots/boxes = thawed sperm, red dots/pink boxes = washed sperm. Time points: 0 = baseline, 1 = immediately after washing/thawing, 2 = 75 min after washing/thawing. Boxes = interquartile range, line = median, whiskers = non‐outlier range. Significant group differences are indicated by brackets (^***^ *p* < 0.001).

Matched‐pairs analysis revealed that sperm vitality decreased significantly in both groups over the measured interval, with thawed sperm samples demonstrating a greater decline in vitality (21 ± 14%) compared to washed samples (7 ± 5%, *p* < 0.0001). Repeated‐measures analysis confirmed this finding, highlighting a significant interaction effect between group type and time, accounting for 90.3% of variance (marginal *R* ^2^ = 0.903, *p* < 0.0001). As illustrated in Figure 2, the decline in vitality follows a similar pattern to that observed with motility, with thawed samples showing accelerated deterioration.

#### 3.1.3 DNA Fragmentation Index

Matched‐pairs analysis indicated an increase in DFI over time in both thawed and washed samples. Statistical significance for this increase over time was observed in the Thawed Group (*p* = 0.0331), whereas the increase in washed samples did not reach significance (*p* = 0.1962). When analyzed as percent change between Time 1 and Time 2, DFI increased by 61 ± 66% in washed samples compared to 65 ± 57% in thawed samples. Although the rate of increase appeared larger in thawed samples, this difference in percent change between groups did not reach statistical significance (*p* = 0.4197).

Table 1 shows that despite similar baseline values, thawed samples exhibited substantially higher absolute DFI values at the final time point. Notably, the mean DFI in thawed samples reached 34 ± 13% at Time 2, exceeding the clinically significant threshold of 30% that is associated with decreased fertility potential, while washed samples remained within normal clinical range (12 ± 5%).

**FIGURE 3:**
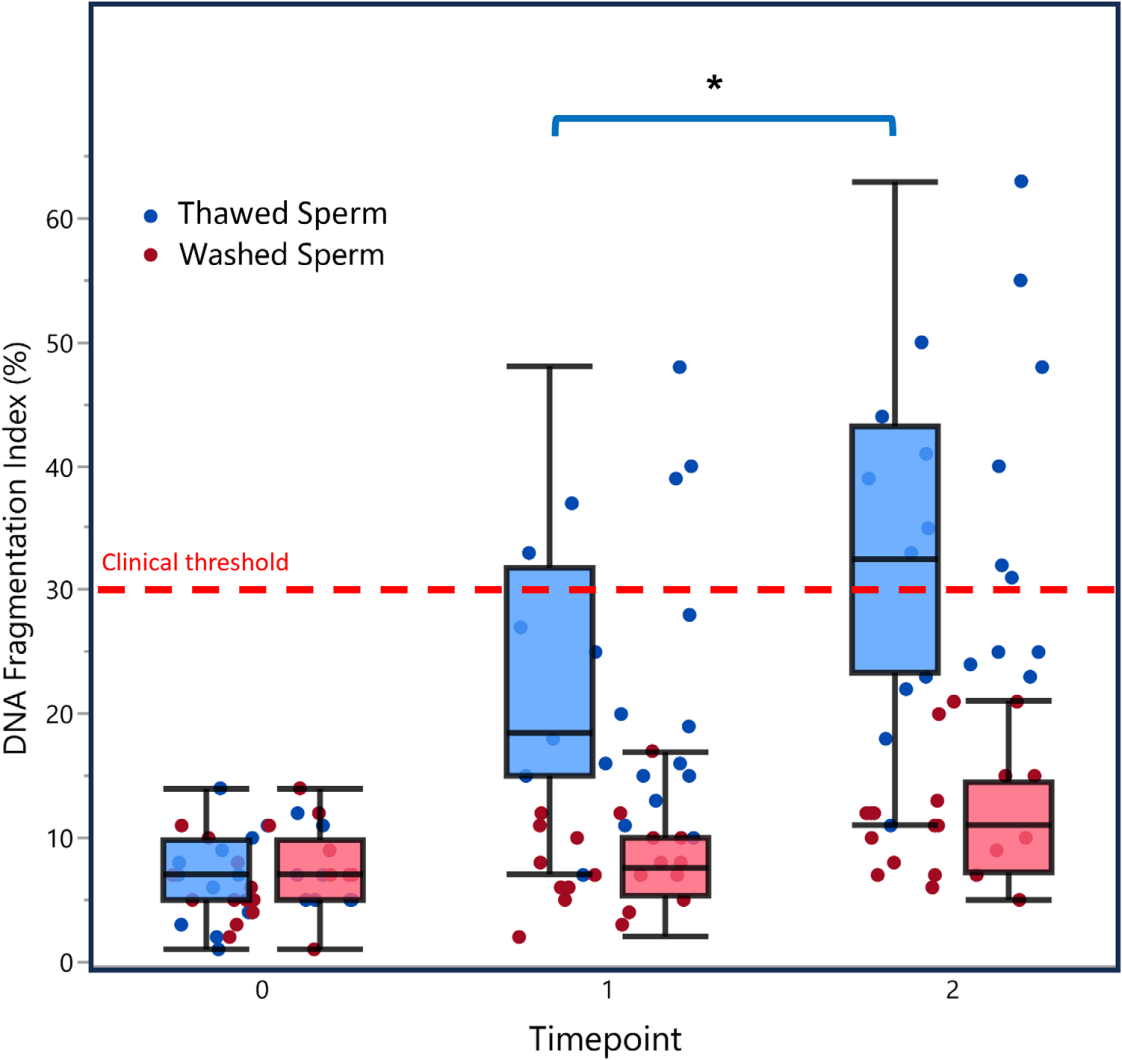
Effect of time on DNA fragmentation index in thawed versus washed sperm samples. Box plots with individual data points (subset *n* = 20). Blue dots/boxes = thawed sperm, red dots/pink boxes = washed sperm. Time points: 0 = baseline, 1 = immediately after washing/thawing, 2 = 75 min after washing/thawing. Dashed red line marks the clinical threshold (30% DFI). Statistical differences between frozen groups are indicated by brackets (^*^ *p* < 0.05).

Repeated‐measures analysis identified a significant interaction between sample type and time, indicating that thawed samples exhibited greater susceptibility to DNA fragmentation over consecutive assessments (marginal *R* ^2^ = 0.870, *p* = 0.0221). Figure 3 illustrates the raw DFI values from Table 1, highlighting the differential impact of time on DNA integrity between the two sample types.

#### 3.1.4 Comparative Analysis of Motility Grade Transitions

Matched‐pairs analysis of motility grades revealed distinct transition patterns between the two groups. When analyzed as percent change, Grade A motility showed similar percentage reductions in both groups (35 ± 17% in washed samples vs. 31 ± 79% in thawed samples, *p* = 0.3840). However, the pathway of this reduction differed significantly. In washed samples, Grade B motility increased by 18 ± 68% (indicated by the negative sign in the context of deterioration analysis, *p* = 0.0170), suggesting that sperm cells transitioned from Grade A primarily to Grade B, thereby retaining some progressive movement. In contrast, in thawed samples, Grade B motility decreased by 7 ± 60% (*p* = 0.0073), indicating that Grade A sperm cells bypassed the intermediate Grade B state and transitioned directly to Grade C or immotility, reflecting a more pronounced decline in functional quality.

Motility Grade C increased in both groups, though the pattern differed significantly. The percent increase was greater in washed samples (107 ± 95%, indicated by the negative sign in deterioration analysis) compared to thawed samples (58 ± 107%, *p* = 0.0089). This pattern is consistent with the observation that in thawed samples, a larger proportion of cells progressed beyond Grade C to complete immotility, resulting in smaller apparent increases in Grade C.

**FIGURE 4:**
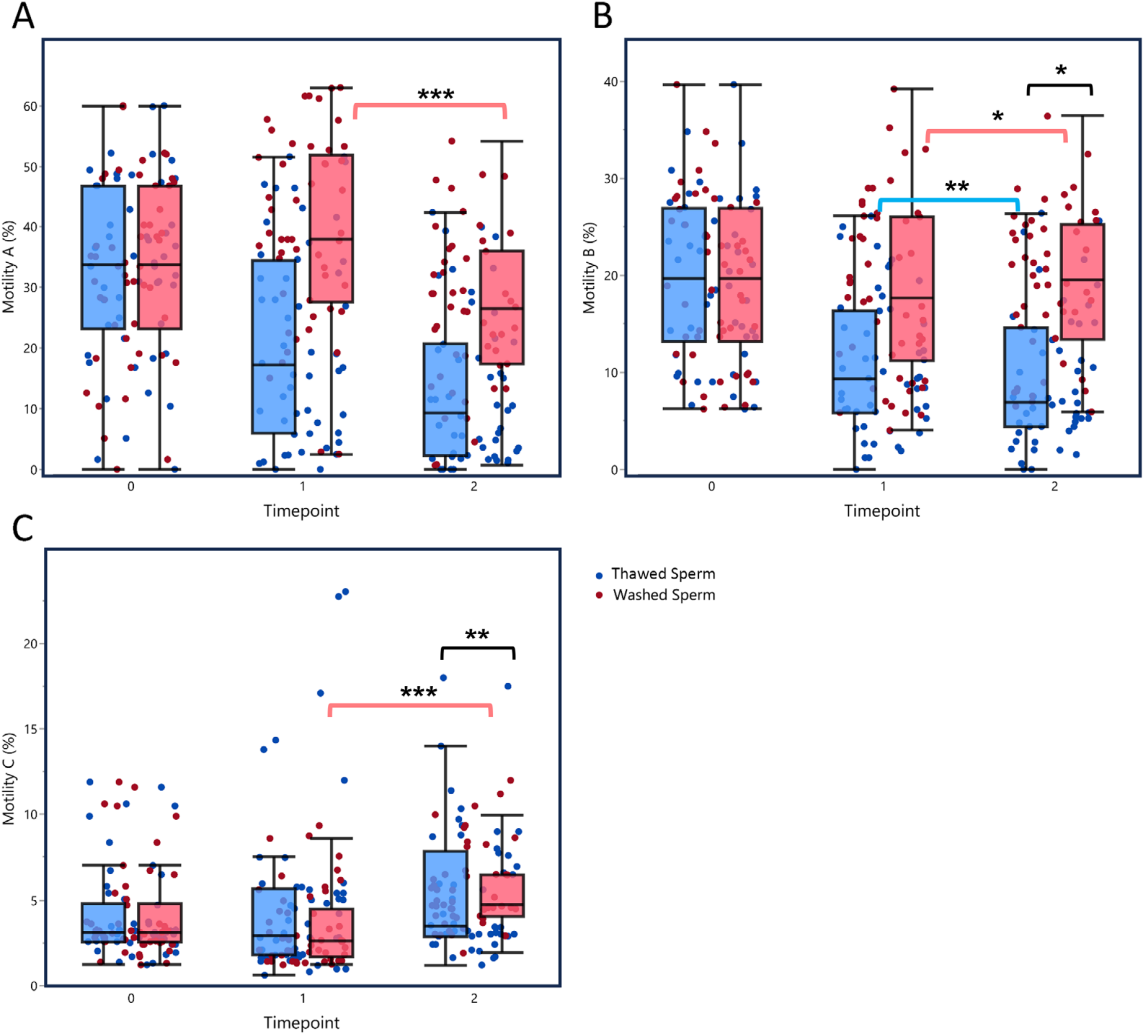
Differential motility grade transitions in thawed versus washed sperm samples. (A) Rapid progressive motility (Grade A), (B) slow progressive motility (Grade B), and (C) non‐progressive motility (Grade C). Blue dots/boxes = thawed sperm, red dots/pink boxes = washed sperm. Time points: 0 = baseline, 1 = immediately after washing/thawing, 2 = 75 min after washing/thawing. Box plots show medians, interquartile range, and whiskers for non‐outlier values. Statistical comparisons are shown by brackets with corresponding significance levels (^*^ *p* < 0.05, ^**^ *p* < 0.01, ^***^ *p* < 0.001).

Repeated‐measures analysis further supported these findings, showing a significant interaction effect between motility grade transition and sperm type, with variance explanations of 87.4% for motility Grade A (*R* ^2^ = 0.874), 80.5% for motility Grade B (*R* ^2^ = 0.805), and 59.9% for motility Grade C (*R* ^2^ = 0.599), *p* < 0.01. These findings emphasize the differential motility transition patterns between groups, where thawed samples are more prone to complete loss of progressive motility. Figure 4 visually summarizes these patterns based on the raw data presented in Table 1.

### 3.2 Effects of Cryoprotectant Exposure and Post‐Thaw Washing on Sperm Quality

Given the prolonged exposure times in our study design, we investigated whether cryoprotectant toxicity might contribute to the observed deterioration. To isolate potential cryoprotectant effects, fresh washed sperm samples from each patient were divided into two equal aliquots: Group A maintained in washing medium alone, and Group B with added freezing medium. Comprehensive comparison revealed no significant differences across all measured parameters. Total motility demonstrated no significant difference (63 ± 10% vs. 63 ± 10% at Time 1; 54 ± 9% vs. 53 ± 10% at Time 2). Progressive motility grades showed no significant differences between groups. Vitality showed minimal non‐significant difference (82 ± 5% vs. 83 ± 6% at Time 1; 77 ± 6% vs. 77 ± 6% at Time 2). DNA fragmentation was comparable between groups at both time points (8 ± 2% vs. 8 ± 2% at Time 1; 9 ± 2% vs. 9 ± 2% at Time 2).

Post‐thaw washing analysis: To determine whether post‐thaw washing affects time‐dependent deterioration, thawed samples from each donor were divided into two equal aliquots: Group C remained as thawed, while Group D was washed immediately post‐thaw to remove cryoprotectant. Total motility showed no significant difference between groups (44 ± 6% vs. 44 ± 6% at Time 1; 33 ± 7% vs. 33 ± 7% at Time 2). Progressive motility grades demonstrated no significant differences between treatments. Vitality demonstrated similar patterns (75 ± 3% vs. 75 ± 3% at Time 1; 62 ± 4% vs. 60 ± 4% at Time 2). DNA fragmentation showed comparable increases in both conditions (19 ± 2% vs. 18 ± 2% at Time 1; 29 ± 2% vs. 30 ± 3% at Time 2).

## 4 Discussion

The results of this investigation demonstrate conclusively that time‐dependent deterioration affects thawed sperm samples substantially more than fresh washed samples. Using a within‐subject design, we documented accelerated decline in motility, vitality, and DNA integrity in thawed samples, with distinctive functional consequences in motility transition patterns.

### 4.1 Mechanisms of Sperm Deterioration: Fresh Versus Thawed Samples

All sperm samples naturally deteriorate over time through several key mechanisms. In fresh samples, this deterioration primarily involves ROS, which play a pivotal role by inducing oxidative stress. This compromises sperm membrane integrity through lipid peroxidation of polyunsaturated fatty acids, particularly affecting the sperm's midpiece where mitochondria are concentrated, disrupting energy production necessary for motility [19, 20]. In addition, ongoing metabolic activity in spermatozoa results in progressive depletion of energy substrates. Sperm cells maintain continuous ATP production through both oxidative phosphorylation and glycolysis to sustain motility. As available nutrients are consumed, energy production becomes compromised, directly impacting sperm kinematic parameters [21].

In thawed samples, these baseline deterioration mechanisms are significantly amplified and supplemented by freeze‐thaw‐specific damage. Physical damage occurs through extracellular ice crystal formation, which affects cellular morphology and concentrates solutes around cells [22]. The intracellular water–cryoprotectant exchange during freezing and thawing leads to harmful cell volume fluctuations that further stress cellular structures. Chemical stress arises from rapid osmolarity changes during freeze‐thawing cycles, causing membrane deformation and additional damage from cryoprotectants like glycerol [23].

The freeze‐thaw process significantly increases oxidative stress through elevated ROS production, resulting in reduced motility and enhanced lipid membrane peroxidation [10]. Mitochondrial damage, frequently observed in correlation with motility loss in thawed sperm, often occurs as part of widespread cellular destruction [24]. DNA integrity is compromised through several pathways: ice crystal formation can disrupt DNA–protein interactions, oxidative stress can damage nuclear DNA while impairing repair mechanisms, and activation of apoptotic‐like cascades can trigger caspase‐dependent endonuclease activity leading to DNA fragmentation [25].

### 4.2 Cryoprotectant Effects

The possibility that cryoprotectants themselves contribute to sperm deterioration has been raised in several studies. In humans, Raad et al. (2018) compared five commercial cryopreservation media, including TEST‐yolk buffer (Medium E in their study), and reported reduced motility, altered morphology, and increased DNA fragmentation, particularly in infertile men. Their experiments were performed on raw, unwashed semen, which retains leukocytes and cellular debris and is therefore more prone to generating ROS, as well as being more susceptible to osmotic stress [26]. In our protocol, spermatozoa were pre‐washed, and the freezing medium was introduced gradually (dropwise) in order to minimize osmotic fluctuations. In a separate line of research, mouse spermatozoa from β‐thalassemia models showed enhanced sensitivity to cryopreservation, with elevated oxidative stress compared to controls [27].

In our setting, the medium effect analysis revealed no differences between aliquots incubated in washing versus freezing medium, indicating that cryoprotectant exposure alone does not accelerate deterioration in human spermatozoa. Moreover, post‐thaw washing to remove cryoprotectant did not improve motility, vitality, or DNA fragmentation. Taken together, these findings indicate that the principal driver of accelerated decline is the freeze‐thaw process itself, rather than ongoing cryoprotectant exposure. This distinction carries important clinical implications, highlighting that laboratory efforts should prioritize minimizing the interval between thawing and insemination rather than additional post‐thaw washing steps.

### 4.3 Novel Contribution and Proposed Mechanism

Our study builds upon previous research by Zaghi et al. (2020), which demonstrated time‐dependent deterioration in thawed sperm. However, while Zaghi's work primarily examined the effects of repeated freeze‐thaw cycles [15], our investigation specifically addresses the differential impact of time on thawed versus fresh washed samples from the same individuals. This within‐subject design eliminates individual variability as a confounding factor, allowing for direct isolation of the time‐dependent effects unique to each sample type.

A key innovation in our work is the detailed analysis of motility grade transitions. While both fresh and thawed samples showed similar percentage reductions in Grade A motility, the downstream fate of these cells differed dramatically. In washed samples, Grade B motility significantly increased, indicating that sperm cells transitioned from Grade A primarily to Grade B, thereby retaining some progressive movement. In contrast, in thawed samples, Grade B motility decreased, showing that Grade A sperm cells bypassed the intermediate Grade B state and transitioned directly to Grade C or immotility.

The DNA fragmentation data further illustrate this differential vulnerability. Thawed samples reached a mean DFI of 34% at Time 2, exceeding the clinically significant threshold of 30% associated with decreased fertility potential, while washed samples remained within normal clinical range (12%). These findings reveal that apparent similarities in overall motility decline between groups mask important differences in the quality and pattern of that decline.

Based on these findings, we propose a cumulative stress model to explain the accelerated deterioration in thawed sperm. In this model, the pre‐existing cellular damage from the freeze‐thaw process substantially reduces the cell's resilience to normal time‐dependent stressors, creating a synergistic negative effect. This model is supported by our vitality measurements, where thawed samples showed a threefold greater decline compared to washed samples. The progressive deterioration appears to follow a threshold effect, where cells already compromised by cryopreservation lack sufficient reserves to withstand additional stressors, leading to rapid functional decline when exposed to identical time conditions.

The direct transition from Grade A to immotility in thawed samples suggests that these cells undergo abrupt rather than gradual deterioration, possibly due to accelerated membrane destabilization, mitochondrial dysfunction, or activation of apoptotic pathways when minimal additional stress is applied to already compromised cells.

### 4.4 Clinical Implications

Our findings have several important clinical applications for assisted reproductive technologies. Based on the accelerated deterioration observed in thawed samples, we recommend establishing specific time‐based guidelines for thawed sperm utilization. Our data show significant deterioration at 75 min post‐thawing, suggesting that insemination procedures should ideally be performed as soon as possible after thawing to preserve optimal functional quality, especially for samples with borderline parameters.

The data suggest potential benefits in performing insemination procedures at sperm bank facilities rather than transporting specimens to external clinics. This approach would eliminate transport‐related quality deterioration and optimize the functional capacity of thawed sperm. For facilities where on‐site insemination is not feasible, strict time limits should be implemented between thawing and insemination procedures.

## 5 Limitations

This study addressed fundamental aspects of sperm cryobiology but did not directly assess clinical outcomes such as pregnancy rates. Future studies should investigate whether the observed accelerated deterioration of thawed sperm translates to reduced pregnancy rates when insemination is delayed.

In addition, our study examined a single cryopreservation protocol with specific time points (immediate post‐processing and 75 min later). Future research could explore whether alternative cryopreservation methods or cryoprotectants might better preserve post‐thaw resilience against time‐dependent deterioration, and establish a more granular timeline with additional intermediate time points to provide further insights into the deterioration curve and potentially identify critical thresholds for clinical applications.

Finally, the control experiments were performed with a relatively small sample size and tested only one cryopreservation medium (TEST‐yolk buffer), which may limit the generalizability of these findings.

## 6 Conclusion

Our study provides compelling evidence that thawed sperm exhibits significantly greater susceptibility to time‐dependent deterioration than fresh washed sperm. The differential patterns of motility loss and DNA fragmentation between the two groups highlight the cumulative damage caused by freezing‐thawing processes and subsequent time‐dependent degradation.

These findings have direct clinical implications for assisted reproductive technologies, particularly intrauterine insemination using thawed sperm. Based on our results, we strongly recommend minimizing the interval between sperm thawing and insemination to optimize sperm functional quality and potentially improve reproductive outcomes.

## Author Contributions

**Adiel Kahana**: conceptualization, investigation, formal analysis, resources, writing – original draft. **Emily Hamilton**: methodology, investigation, data curation, writing – review and editing. **Noga Fuchs Weizman**: formal analysis, validation, writing – review and editing. **Sandra E. Kleiman**: validation, writing – review and editing. **Shlomit Shabat**: methodology, investigation, data curation. **Foad Azem**: supervision, resources, validation. **Shimi Barda**: conceptualization, methodology, formal analysis, project administration, supervision, writing – original draft, writing – review and editing.

## Funding

The authors have nothing to report.

## Conflicts of Interest

The authors declare no conflicts of interest.

## Supporting information




**Supporting File 1**: andr70150‐sup‐0001‐Appendix.docx.

## Data Availability

The data that support the findings of this study are available from the corresponding author upon reasonable request.

## References

[1] J. K. Sherman , “Synopsis of the Use of Frozen Human Semen Since 1964: State of the Art of Human Semen Banking,” Fertility and Sterility 24, no. 5 (1973): 397–412. 4735423 10.1016/s0015-0282(16)39678-9

[2] V. Isachenko , R. Maettner , A. M. Petrunkina , et al., “Cryoprotectant‐Free Vitrification of Human Spermatozoa in Large (up to 0.5 mL) Volume: A Novel Technology,” Clinical Laboratory 57, no. 9–10 (2011): 643–650. 22029178

[3] M. Hezavehei , M. Sharafi , H. M. Kouchesfahani , et al., “Sperm Cryopreservation: A Review on Current Molecular Cryobiology and Advanced Approaches,” Reproductive Biomedicine Online 37, no. 3 (2018): 327–339, 10.1016/j.rbmo.2018.05.012. 30143329

[4] S. R. Soares , M. Cruz , V. Vergara , A. Requena , and J. A. García‐Velasco , “Donor IUI Is Equally Effective for Heterosexual Couples, Single Women and Lesbians, but Autologous IUI Does Worse,” Human Reproduction 34, no. 11 (2019): 2184–2192. 31711203 10.1093/humrep/dez179

[5] J. T. Anger , B. R. Gilbert , and M. Goldstein , “Cryopreservation of Sperm: Indications, Methods and Results,” Journal of Urology 170, no. 4 pt. 1 (2003): 1079–1084, 10.1097/01.ju.0000084820.98430.b8. 14501696

[6] A. Makler , I. Zaidise , E. Paldi , and J. M. Brandes , “Factors Affecting Sperm Motility. I. In Vitro Change in Motility With Time After Ejaculation,” Fertility and Sterility 31, no. 2 (1979): 147–154, 10.1016/s0015-0282(16)43815-x. 761677

[7] N. Chomsrimek , W. Choktanasiri , A. Wongkularb , and O. Prasertsawat , “Effect of Time Between Ejaculation and Analysis on Sperm Motility,” Thai Journal of Obstetrics and Gynaecology 16, no. 2 (2008): 109–114.

[8] H. Mossad , M. Morshedi , J. P. Toner , and S. Oehninger , “Impact of Cryopreservation on Spermatozoa From Infertile Men: Implications for Artificial Insemination,” Archives of Andrology 33, no. 1 (1994): 51–57, 10.3109/01485019408987802. 7979809

[9] J. Yuan , “Protein Degradation and Phosphorylation After Freeze Thawing Result in Spermatozoon Dysfunction,” Proteomics 14, no. 2–3 (2014): 155–156, 10.1002/pmic.201300564. 24382660

[10] L. K. Thomson , S. D. Fleming , K. Barone , J. A. Zieschang , and A. M. Clark , “The Effect of Repeated Freezing and Thawing on Human Sperm DNA Fragmentation,” Fertility and Sterility 93, no. 4 (2010): 1147–1156, 10.1016/j.fertnstert.2008.11.023. 19135665

[11] M. K. Samplaski , A. Dimitromanolakis , K. C. Lo , et al., “The Relationship Between Sperm Viability and DNA Fragmentation Rates,” Reproductive Biology and Endocrinology 13, no. 1 (2015): 1–6. 25971317 10.1186/s12958-015-0035-y PMC4432573

[12] J. Ribas‐Maynou , A. Garcia‐Peiro , A. Fernandez‐Encinas , et al., “Comprehensive Analysis of Sperm DNA Fragmentation by Five Different Assays: TUNEL Assay, SCSA, SCD Test and Alkaline and Neutral Comet Assay,” Andrology 1, no. 5 (2013): 715–722, 10.1111/j.2047-2927.2013.00111.x. 23843251

[13] G. M. Bareh , E. Jacoby , P. Binkley , T. Chang , R. S. Schenken , and R. D. Robinson , “Sperm DNA Fragmentation in Normozoospermic Male Partners of Couples With Unexplained Recurrent Pregnancy Loss,” Fertility and Sterility 105, no. 2 (2016): 329–336. 26607021 10.1016/j.fertnstert.2015.10.033

[14] F. Amidi , A. Pazhohan , M. Shabani Nashtaei , M. Khodarahmian , and S. Nekoonam , “The Role of Antioxidants in Sperm Freezing: A Review,” Cell and Tissue Banking 17, no. 4 (2016): 745–756, 10.1007/s10561-016-9566-5. 27342905

[15] B. Zaghi , S. Barda , S. Kleiman , and R. Hauser , “Impact of Time Between Repeated Sperm Freezing Cycles on Sperm Quality,” Reproductive Biology 20, no. 1 (2020): 75–80. 31879229 10.1016/j.repbio.2019.12.003

[16] J. I. Campbell and V. A. Thompson , “MorePower 6.0 for ANOVA With Relational Confidence Intervals and Bayesian Analysis,” Behavior Research Methods 44, no. 4 (2012): 1255–1265, 10.3758/s13428-012-0186-0. 22437511

[17] World Health Organization . WHO Laboratory Manual for the Examination and Processing of Human Semen, 6th ed. (World Health Organization, 2021).

[18] T. F. Kruger , A. A. Acosta , K. F. Simmons , R. J. Swanson , J. F. Matta , and S. Oehninger , “Predictive Value of Abnormal Sperm Morphology in In Vitro Fertilization,” Fertility and Sterility 49, no. 1 (1988): 112–117, 10.1016/s0015-0282(16)59660-5. 3335257

[19] A. Agarwal , G. Virk , C. Ong , and S. S. du Plessis , “Effect of Oxidative Stress on Male Reproduction,” World Journal of Men's Health 32, no. 1 (2014): 1–17, 10.5534/wjmh.2014.32.1.1. PMC4026229 24872947

[20] R. J. Aitken and M. A. Baker , “Causes and Consequences of Apoptosis in Spermatozoa; Contributions to Infertility and Impacts on Development,” International Journal of Developmental Biology 57, no. 2–4 (2013): 265–272, 10.1387/ijdb.130146ja. 23784837

[21] W. C. L. Ford , “Glycolysis and Sperm Motility: Does a Spoonful of Sugar Help the Flagellum Go Round?” Human Reproduction Update 12, no. 3 (2006): 269–274. 16407453 10.1093/humupd/dmi053

[22] J. Morris , E. Acton , B. J. Murray , and F. Fonseca , “Freezing Injury: The Special Case of the Sperm Cell,” Cryobiology 64, no. 2 (2012): 71–80. 22197768 10.1016/j.cryobiol.2011.12.002

[23] M. Di Santo , N. Tarozzi , M. Nadalini , and A. Borini , “Human Sperm Cryopreservation: Update on Techniques, Effect on DNA Integrity, and Implications for ART,” Advances in Urology 2012, no. 1 (2012): 854837, 10.1155/2012/854837. 22194740 PMC3238352

[24] M. O'Connell , N. McClure , and S. E. Lewis , “The Effects of Cryopreservation on Sperm Morphology, Motility and Mitochondrial Function,” Human Reproduction 17, no. 3 (2002): 704–709, 10.1093/humrep/17.3.704. 11870124

[25] H. Amor , A. Zeyad , Y. Alkhaled , et al., “Relationship Between Nuclear DNA Fragmentation, Mitochondrial DNA Damage and Standard Sperm Parameters in Spermatozoa of Fertile and Sub‐Fertile Men Before and After Freeze‐Thawing Procedure,” Andrologia 50, no. 5 (2018): e12998. 29527711 10.1111/and.12998

[26] G. Raad , L. Lteif , R. Lahoud , et al., “Cryopreservation Media Differentially Affect Sperm Motility, Morphology and DNA Integrity,” Andrology 6, no. 6 (2018): 836–845, 10.1111/andr.12531. 30105872

[27] K. Buranaamnuay , S. Aiemongkot , C. Changsangfa , and S. Svasti , “The Effect of Cryopreservation Media on the Quality of β‐Thalassemia Mouse Spermatozoa,” Open Veterinary Journal 12, no. 5 (2022): 602–611, 10.5455/OVJ.2022.v12.i5.2. 36589404 PMC9789754

